# Machine Learning in Action: Stroke Diagnosis and Outcome Prediction

**DOI:** 10.3389/fneur.2021.734345

**Published:** 2021-12-06

**Authors:** Shraddha Mainali, Marin E. Darsie, Keaton S. Smetana

**Affiliations:** ^1^Department of Neurology, Virginia Commonwealth University, Richmond, VA, United States; ^2^Department of Emergency Medicine, University of Wisconsin Hospitals and Clinics, Madison, WI, United States; ^3^Department of Neurological Surgery, University of Wisconsin Hospitals and Clinics, Madison, WI, United States; ^4^Department of Pharmacy, The Ohio State University Wexner Medical Center, Columbus, OH, United States

**Keywords:** machine learning, artificial intelligence, deep learning, stroke diagnosis, stroke prognosis, stroke outcome prediction, machine learning in medical imaging, machine learning in medicine

## Abstract

The application of machine learning has rapidly evolved in medicine over the past decade. In stroke, commercially available machine learning algorithms have already been incorporated into clinical application for rapid diagnosis. The creation and advancement of deep learning techniques have greatly improved clinical utilization of machine learning tools and new algorithms continue to emerge with improved accuracy in stroke diagnosis and outcome prediction. Although imaging-based feature recognition and segmentation have significantly facilitated rapid stroke diagnosis and triaging, stroke prognostication is dependent on a multitude of patient specific as well as clinical factors and hence accurate outcome prediction remains challenging. Despite its vital role in stroke diagnosis and prognostication, it is important to recognize that machine learning output is only as good as the input data and the appropriateness of algorithm applied to any specific data set. Additionally, many studies on machine learning tend to be limited by small sample size and hence concerted efforts to collate data could improve evaluation of future machine learning tools in stroke. In the present state, machine learning technology serves as a helpful and efficient tool for rapid clinical decision making while oversight from clinical experts is still required to address specific aspects not accounted for in an automated algorithm. This article provides an overview of machine learning technology and a tabulated review of pertinent machine learning studies related to stroke diagnosis and outcome prediction.

## Introduction

The term machine learning (ML) was coined by Arthur Samuel in 1959 (1). He investigated two machine learning procedures using the game of checkers and concluded that computers can be programmed quickly to play a better game of checkers than the person who wrote the program. Simply put, machine learning can be defined as a subfield of artificial intelligence (AI) that uses computerized algorithms to automatically improve performance through iterative learning process or experience (i.e., data acquisition) (2). Of late, the field of ML has vastly evolved with the development of various computerized algorithms for pattern recognition and data assimilation to improve predictions, decisions, perceptions, and actions across various fields and serves as an extension to the traditional statistical approaches. In our day-to-day life, a relatable example of ML is the application of spam filters to the 319 billion emails sent and received daily worldwide, of which, nearly 50% can be classified as spam (3). Use of ML technology has made this process efficient and manageable. The ML technology utilizes various methods for automated data analysis including linear and logistic regression models as well as other methods such as the support vector machines (SVM), random forests (RF), classification trees and discriminant analysis that allow combination of features (data points) in a non-linear manner with flexible decision boundaries. The advent of neural networks and deep learning (DL) technology has transformed the field of ML with automatic and efficient feature identification and processing within a covert analytic network, without the need for a priori feature selection. Notably, performance of DL is known to improve with access to larger datasets, whereas classic ML methods tend to plateau at relatively lower performance levels. Hence, in this era of big data where clinicians are constantly inundated with plethora of clinical information, use of DL technology has significnalty enhanced our ability to assimilate the vast amount of clinical data to make expeditious clinical decision.

Stroke is a leading cause of death, disability, and cognitive impairment in the United States (4). According to the 2013 policy statement from the American Heart Association, an estimated 4% of US adults will suffer from a stroke by 2030, accounting for total annual stroke-related medical cost of $240.67 billion by 2030 (5). For ischemic stroke, acute management is highly dependent on prompt diagnosis. According to the current ischemic stroke guidelines, patients are eligible for intravenous thrombolysis up to 4.5 h from symptom onset and endovascular thrombectomy without advanced imaging within 6 h of symptom onset (6–8). For patients presenting between 6 and 24 h of symptom onset (or last known well time), advanced imaging is recommended to assess salvageable penumbra for decisions regarding endovascular therapy (9–11). Similarly for hemorrhagic stroke, timely diagnosis utilizing imaging technology to evaluate the type and etiology of hemorrhage is important in guiding acute treatment decisions. Prompt diagnosis with emergent treatment decision and accurate prognostication is hence the cornerstone of acute stroke management. Over the recent years, a multitude of ML methodologies have been applied to stroke for various purposes, including diagnosis of stroke (12, 13), prediction of stroke symptom onset (14, 15), assessment of stroke severity (16, 17), characterization of clot composition (18), analysis of cerebral edema (19), prediction of hematoma expansion (20), and outcome prediction (21–23). In particular, there has been a rapid increase in the trend of ML application for *imaging-based* stroke diagnosis and outcome prediction. The Ischemic Stroke Lesion Segmentation Challenge (ISLES: http://www.isles-challenge.org/) provides a global competing platform encouraging teams across the world to develop advanced tools for stroke lesion analysis using ML. In this platform, competitors train their algorithms on a standardized dataset and eventually generate benchmarks for algorithm performance.

**Figure 1:**
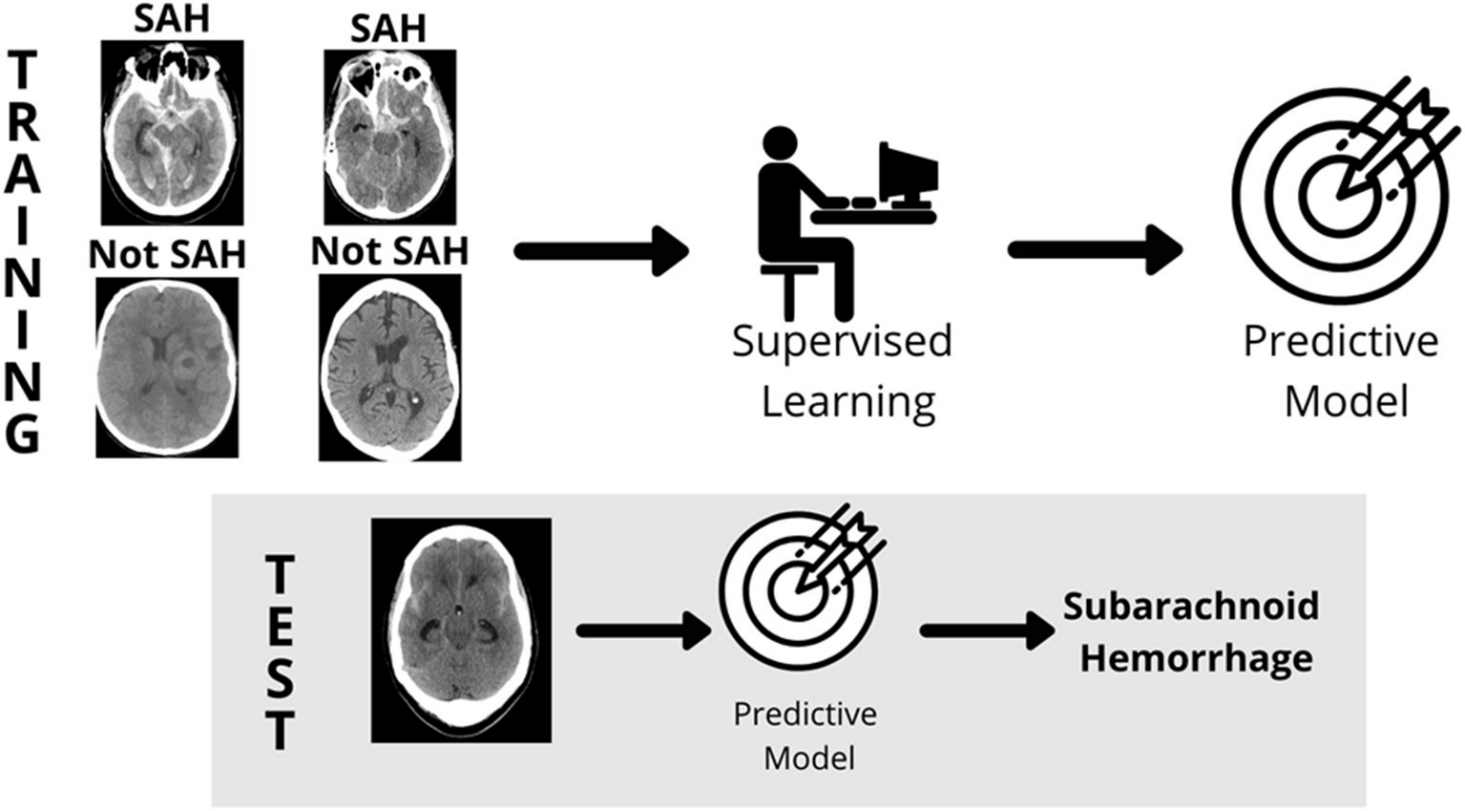
Supervised learning. In supervised learning, a model is built by labeling images [Subarachnoid Hemorrhage (SAH) and Not Subarachnoid Hemorrhage (Not SAH)], a predictive model is created, and then tested for accuracy in reading unlabeled images (gray box). Source: WesternDigital BLOG.

**Figure 2:**
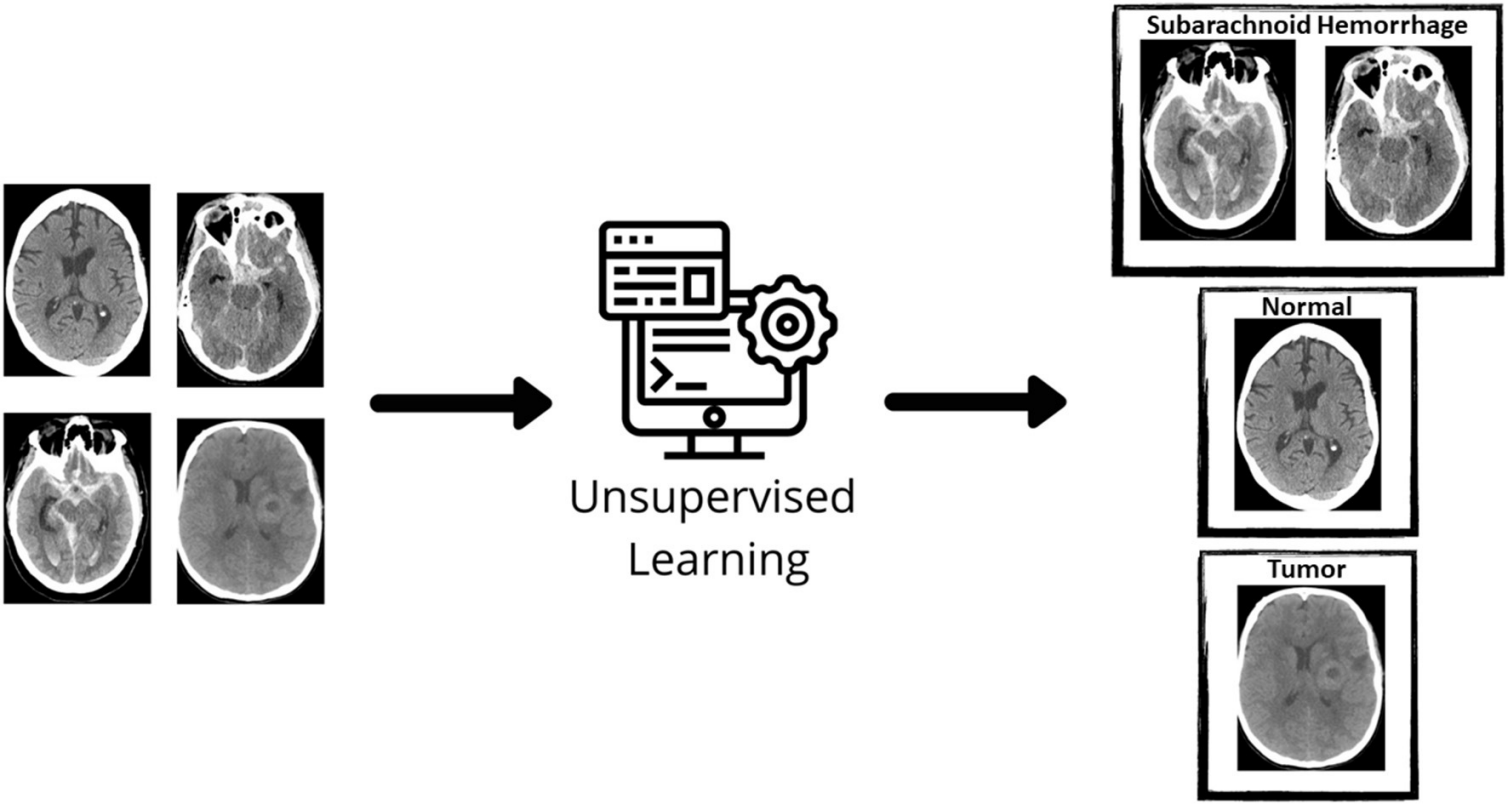
Unsupervised learning. In unsupervised learning, the machine learning algorithm discovers structures within given data. The initial data is not labeled and a clustering algorithm groups unlabeled data together. Source: WesternDigital BLOG.

**Figure 3:**
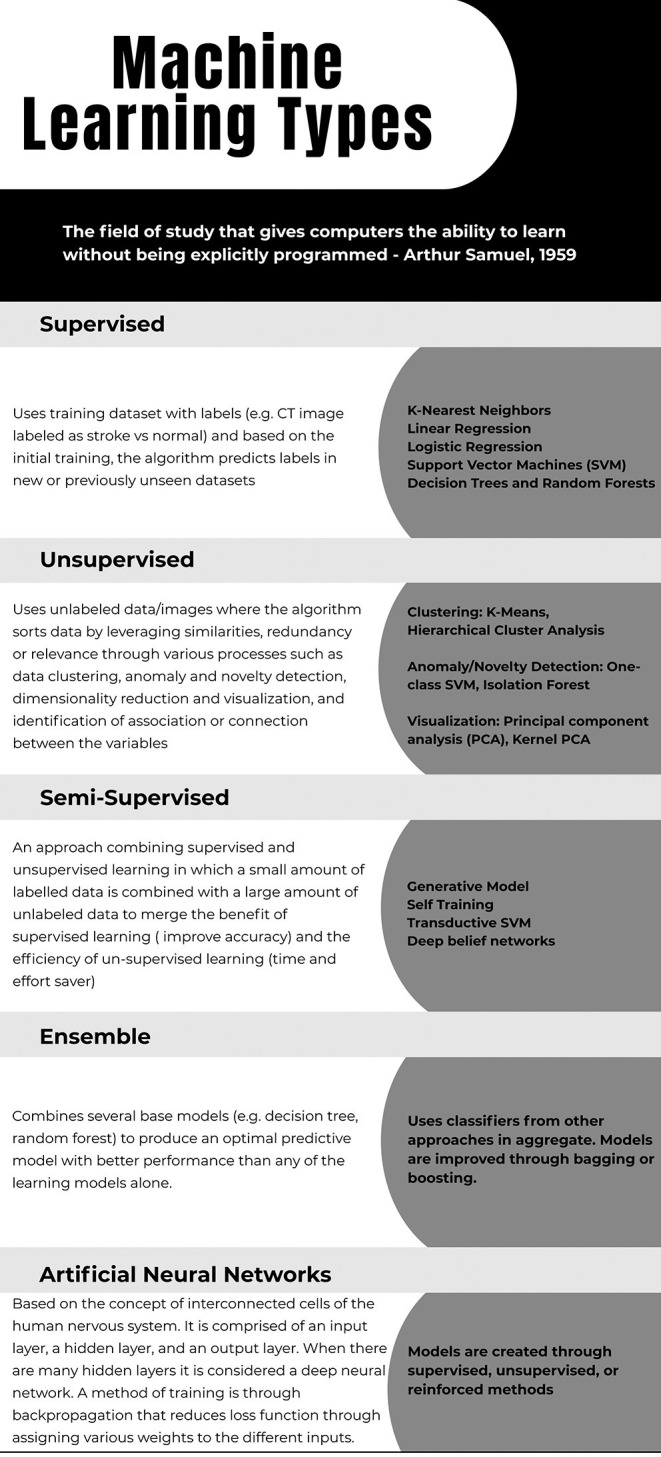
Created from the following referenes: Dey (28) Zhou (29) Geron (30).

Deciding which type of ML to use on a specific dataset depends on factors such as the size of dataset, need for supervision, ability to learn, and the generalizability of the model (24). DL technology such as the deep neural networks has significantly improved the ability for image segmentation, automated featurization (e.g., conversion of raw signal into clinically useful parameter), and multimodal prognostication in stroke; and it is increasingly utilized in stroke-based applications (25–27). For example, DL algorithms can be applied to extract meaningful imaging features for image processing in an increasing order of hierarchical complexity to make predictions, such as the final infarct volume (27). Some commonly used ML types with their respective algorithms and practical examples are outlined in Figures 1–3. In the healthcare setting, supervised and unsupervised algorithms are both commonly used. In this review, we will specifically focus on ML strategies for stroke diagnosis and outcome prediction. Table 1 provides an overview of pertinent studies with use of ML in stroke diagnosis (Section A) and outcome prediction (Section B). A glossary of machine learning terms with brief description is separately provided in Supplementary Table 1.

**Table 1:** Studies utilizing machine learning for stroke diagnosis and prediction.

**References**	**Study objective**	**ML-based approaches**	**Validation method**	**Sample size**	**Feature**	**Optimal results**	**Optimal ML approach**	**Clinical implications**	**Limitations**
**Section A: stroke diagnosis**									
**Ischemic stroke**									
Garca-Terriza et al. (31)	Stroke type diagnosis and mortality	RF	10-fold cross validation resampling	•119 •(AIS 105, ICH 14)	•Type of stroke •Mortality •Non-invasive variables (cardiac and pulmonary)	•*Accuracy* •Subtype - 92% •Mortality - 96%	-	May predict the type of stroke a patient is at risk for and outcomes	Data obtained after event to for prediction models but do not include usual risk factors for consideration
Sung et al. (32)	Ischemic stroke phenotype^*^	Various models (C4.5, CART, KNN, RF, SVM, LR, with aggregation algorithms	10-fold cross validation	4,640	Clinical notes with preprocessing and MetaMap to identify medical entities +/- NIHSS	•*Accuracy; kappa* •NIHSS + text •(0.489–0.583; 0.272–0.399) •NIHSS •(0.465–0.533; 0.254–0.344) •Text •(0.465–0.533; 0.170–0.328)	-	Clinical text plus validated scoring tools might aid in phenotyping of stroke	•Phenotype based on OCSP definitions,^*^ •Difficult delineating certain phenotypes, •Unclear who were the authors of the clinic notes
Giri et al. (33)	Ischemic stroke diagnosis by EEG	1D CNN vs. various models (NB, Classification Tree, ANN, RF, kNN, LR)	Leave-one-out cross-validation	•32 – AIS •30 – Controls	15-min EEG with 24 chosen features	•Accuracy - 0.86 •F-Score 0.861	Leave-one-out scenario of 1D CNN	In areas with limited access to CT imaging may help diagnosis AIS	Time to apply EEG electrodes may result in delays of care
Lee et al. (14)	Identify patients within 4.5-h thrombolysis window	LR, RF, SVM	•85% training •15% test	355	MRI features	•Sensitivity 75.8% •Specificity 82.6% •AUC 85.1%	RF	Improved sensitivity than human readings in identifying stroke patients within thrombolysis window	Assessed only dichotomized visibility of signals in the lesion territory
Ho et al. (15)	Classifying onset time from imaging	LR, RF, GBRT, SVM, SMR	10-fold cross validation on training data with optimal hyperparameters	104	MRI	•Sensitivity 78.8% •AUC 76.5%	LR with deep autoencoder features	Improved stroke onset detection compared to DWI-FLAIR	Trained on MRI only
Takahashi et al. (34)	Detection for MCA dot sign in unenhanced CT	SVM	Not described	297 images	Unenhanced CT	Sensitivity 97.5%	SVM	Accurately detect hyperdense MCA dot sign	Data from 7 patients
Chen et al. (35)	Automatically segment stroke lesions in DWI	CNN	Train / Test	741 subjects	DWI	Dice score 0.67	CNN	Segment stroke lesions automatically	Improved Dice scores on larger lesions
Bouts et al. (36)	Depict ischemic tissue that can recover after reperfusion	GLM, GAM, SVM, Adaptive boosting, RF	Generalized cross validation with unbiased risk estimator scoring	19 rats	MRI	Dice Score 0.79	GLM	MRI-based algorithms could estimate extent of salvageable tissue	Varying efficacy in differentiating between areas irreversibly damaged vs. salvaged after reperfusion
Chen et al. (37)	Quantify cerebral edema following infarction *via* CSF quantification	RF with geodesic active contour segmentation	•10-fold cross validation •Train / Test	38 subjects	CT Imaging	•Baseline Dice Score 0.76 •6-h Dice score 0.73	RF with geodesic active contour segmentation	Efficiently and accurately measure evolution of cerebral edema	
Colak et al. (38)	Stroke Prediction	MLP ANN and SVM with radial basis function kernel	Train / Test	297 subjects (130 sick and 167 healthy)	9 predictors (CAD, DM, HTN, CVA history, AF, smoking, carotid Doppler findings, cholesterol, CRP	•Accuracy 85.9% •AUC 0.93	ANN	Ability to screen patients at risk for stroke based on comorbidities	Factors used to predict model are known to be risk factors for stroke
Maier et al. (39)	Classify lesion segmentation	KNN, GNB, GLM, RF, CNN	Leave-one-out cross-validation	37 subjects	MRI	•RF: •Precision 82% •Recall 62% •CNN: •Precision 77% •Recall 64%	•RF •CNN	Future work may be able to segment lesions	No methods achieved results in the range of the human observer agreement
Öman et al. (40)	Detection of ischemic stroke	3D CNN	Train / Test	60 subjects	CT Angiography	•Sensitivity 93% •Specificity 82% •AUC 0.93 •Dice 0.61	3D CNN	Lesion can be detected with CNN	Contralateral hemisphere data may reduce false positive findings
Chen et al. (41)	Prehospital detection of large vessel occlusion	ANN	10-fold cross validation	600 subjects	Baseline demographics, medical history, NIHSS, risk factors	•Youden index 0.640 •Sensitivity 0.807 •Specificity 0.833 •Accuracy 0.822	ANN	Known patient risk factors may help in predicting large vessel occlusion	Cohort included stroke patients and not those with mimics or hemorrhagic stroke
**Hemorrhagic stroke**									
Dhar et al. (42)	Hemorrhage and perihematomal edema (PHE) quantification	CNN	•10-fold cross validation •Train / Test	124	24-h CT head scans	•Dice score •0.9 – hemorrhage •0.54 - PHE	-	Rapid and consistent measurements of supratentorial ICH	-IVH not delineated from ICH
Arab et al. (43)	Hematoma segmentation and volume quantification	CNN with deep supervision based on reader labeling	Train / Test	55	64 axial slices of 128 × 128 voxels	•Dice score •0.84 ± 0.06 •Precision •0.85 ± 0.07 •Recall •0.83 ± 0.07 •F-Score 0.84	CNN with deep supervision	Fast and reliable quantification of hematoma volume	•False positives observed with calcifications •False negatives observed with blood close to bone
Ko et al. (44)	ICH detection	CNN and long-short term memory	Train / Test	5,244,234	Pre-processed CTH to balance subtypes and window settings	•Classification accuracy •92 – 93%	-	Identification of ICH and subtypes	-Preprocessing of data required to attain accuracy
Irene et al. (45)	ICH segmentation and volume approximation	Dynamic Graph CNN	•4-fold cross validation •Train / Test	27	CTH	•Accuracy 96.4% •Precision 0.93 •Recall 0.98 •*F*-Score 0.96	SVM method with radial basis function kernel	Identification of ICH and blood volume prediction	Small dataset
Arbabshinrani et al. (13)	Diagnose ICH and prioritize radiology worklists	Deep CNN	•Training (75%) •Cross validation (5%) •Testing (20%)	46,573 studies	Preprocessing of CTH images	•ROC 0.846 •Specificity 0.8 •Sensitivity 0.73	-	Assist in upgrading image reads to “stat” from “routine”	Did not identify location of ICH
Sage et al. (46)	ICH subtype detection	Double-branch CNN of SVM, RF	Concatenation of double-branch features and classification	9,997 subjects	372,556 images (11,454 CT scans)	•Accuracy range •SVM •76.9 – 96% •RF •74.3 – 96.7%	-	Identify and classify ICH	EDH performed the worst in SVM and RF possibly due to under representation in data
Ye et al. (47)	ICH subtype detection	3D joint CNN – recurrent NN	•Training (80%) •Validation (10%) •Testing (10%)	2,836 subjects	76,621 slices from non-contrast head CT scans	•AUC for +/- ICH • 0.98 •AUC range for subtypes •0.89 – 0.96	-	Identify and classify ICH	SAH classification may have been more difficult due to blended ICH examples
Chang et al. (48)	ICH detection and volume measurements	Hybrid 3D/2D CNN	5-fold cross validation	10,841 Scans	Non-contrast CTH	•*ICH detection* •Accuracy 0.97 •Sensitivity 0.951 •Specificity 0.073 •Volume •Dice score 0.772–0.931	-	Identification of ICH and blood volume prediction	Generalization needs to be confirmed in other institutions
**Subarachnoid hemorrhage**									
Capoglu et al. (49)	Vasospasm prediction	Sparse dictionary learning and covariance-based features	Not described	20	3D brain angiograms	ROC 0.93	-	Proof of concept to predict those who might have vasospasm	Small dataset
Ramos et al. (22)	DCI Prediction	LogReg, SVM, RF, MLP	Monte-Carlo cross-validation with 100 random splits (75% training / 25% test) and 5-fold cross-validation	317	Non-contrast CT image data and 48 clinical variables	•ROC 0.74 •Specificity 0.67 •Sensitivity 0.75	RF with clinical variables and image features	ML improved prediction of DCI especially when image features included (aneurysm height / width)	Manual extraction of features from medical images is time-consuming
Tanioka et al. (50)	DCI prediction	RF	Leave-one-out cross-validation	95	Clinical variables and matricellular proteins (MCP) on days 1 – 3	•Accuracy •93.9% - clinical variables •87.2% - MCP only •95.2% - clinical variables + MCP	-	MCP might play a role in predicting DCI but further data needed	Other biomarkers not assessed
**Miscellaneous**									
Ni et al. (12)	Stroke Case Detection	LR, SVM-P, SVM-R, RF, ANN	Two iterations of 10-fold cross validation	8,131	Medical record information compared to ICD codes	•Accuracy 88.6% •Precision 93.8% •Recall 92.8% •*F* Score 93.3% •AUC 89.8% •AUC-PR 97.5%	RF	Detection of stroke diagnosis through EHR data that was miscoded	Accurate ICD codes limit utility of the algorithm
Park et al. (16)	Autonomously grade NIHSS and MRC scores through wearable sensors	•SVM •Ensemble	5-fold cross validation searched by Bayes optimization in 30 trials	240	Wearable sensors	•NIHSS: •Accuracy 83.3% •AUC 0.912 •MRC: •Accuracy 76.7% AUC 0.87	SVM	Automatic grading in real time of proximal weakness	Requires sensors to be applied
**Section B: stroke outcome prediction**									
**References**	**Study objective**	**ML-based approach**	**Validation method**	**Sample size**	**Feature**	**Optimal results**	**Best predictors**	**Clinical implications**	**Limitations**
**Radiological outcomes**									
Nielsen et al. (26)	Prediction of final infarct volume	CNN_deep_	85% training/15% testing	222	MRI images	AUC 0.88 ± 0.12	-	Facilitates treatment selection	No external validation, retrospective
Giacalone et al. (51)	Prediction of final infarct volume	SVM	K-fold cross-validation	4	MRI images	95% accuracy	-	“ ”	Small sample size, Retrospective
Grosser et al. (52)	Prediction of final infarct volume	XGBoost	Leave-one-out cross-validation	99	MRI images	AUC 0.893 ± 0.085	Spatial lesion probability	“ ”	Retrospective, Limited generalizability (patient data is from 2006 to 2009)
Foroushani et al. (53)	Prediction of malignant cerebral edema	LR	10-fold cross-validation	361	Serial, quantitative CT images	AUC 0.96	Reduction in CSF volume	“ ”	No external validation
Bentley et al. (23)	Prediction of sICH	SVM	K-fold cross-validation	116	Unenhanced CT images	AUC 0.744	Baseline NIHSS, CT evidence of acute ischemia	“ ”	Image processing took ~30 min; Small number of sICH cases
Yu et al. (54)	Prediction of HT	SR-KDA	Leave-one-out cross-validation	155	MRI images	83.7 ± 2.6% accuracy	-	“ ”	Single-center, Retrospective
Scalzo et al. (55)	Prediction of HT	SR-KDA	10-fold cross-validation	263	MRI images	88% accuracy	-	“ ”	Retrospective, current limitations in measuring BBB permeability
van Os et al. (56)	Prediction of reperfusion after EVT (mTICI <2b vs. ≥2b)	LR (using backward elimination)	Nested cross-validation, consisting of an outer and an inner cross-validation loop	1,383	EHR data, CT/CTA images	AUC 0.57	-	“ ”	Retrospective; Only moderate predictive value, LR outperformed machine-learning
Hilbert et al. (57)	Prediction of reperfusion after EVT (mTICI <2b vs. ≥2b)	RFNN-ResNet-AE fine-tuned	4-fold cross-validation	1301	CTA images	Average AUC 0.65	-	“ ”	Retrospective; Only moderate predictive value
Rondina et al. (58)	Comparison of imaging approaches (lesion load per ROI vs. pattern of voxel) to predict post stroke motor impairment	GPR	10-fold cross-validation	50	Post stroke MRI	Best prediction was obtained using motor ROI and CST (derived from probabilistic tractography) R = 0.83, RMSE = 0.68	Patterns of voxels representing lesion probability produced better results	Informs appropriate methodology for predicting long term motor outcomes from early post-stroke MRI.	Small sample size, no external validation
**Discrete morbidity and mortality clinical outcomes**									
Matsumoto et al. (59)	Prediction of all-cause, in-hospital mortality	LASSO	10-fold cross-validation	4,232	EHR data	AUC 0.88	-	Facilitates GOC decision making	Retrospective, Single-center, Limited generalizability (ETV used in only 1.5% of patients), Low rate (3.5%) of in-hospital mortality
Scrutinio et al. (60)	Prediction of 3-yr mortality after severe stroke	SMOTE RF	10-fold cross-validation	1,207	EHR data	AUC 0.928	Age	Facilitates GOC decision making	No external validation
Ge et al. (61)	Prediction of SAP at 7 and 14 d	Attention-augmented GRU	10-fold cross-validation	13,930	EHR data	•7 d: AUC 0.928 •14 d: AUC 0.905	PPI use	Facilitates early detection and targeted application of prophylaxis interventions	Single-center, No external validation
Li et al. (62)	Prediction of SAP at 7 d	XGBoost	5-fold cross-validation	3,160	EHR data	AUC 0.841	Age, Baseline NIHSS, FBG, sex, Premorbid mRS score, & History of AF	“ “	Single-center, No external validation
Wang et al. (63)	Predicting functional outcome (mRS) at 1st and 6th months	RF	10-fold cross-validation	333	Demographics, labs, CT brain	•1 month outcome: AUC 0.899; •6 months outcome AUC: 0.917	•1 month outcome= 26 attributes; •6 months outcome: 22 attributes	Use of ML to predict functional outcome after ICH is feasible, and RF model provides the best predictive performance	Small sample size, excluded large hematomas, did not evaluate hematoma or edema expansion, no external validation
**Functional outcomes**									
Heo et al. (64)	Prediction of mRS score (0–2 vs. 3–6) at 90 d	Deep neural network	67% training/ 33% testing	2,604	EHR data	AUC 0.888	-	Informs patient expectations, Facilitates GOC decision making	Single-center, No external validation
Lin et al. (65)	Prediction of mRS score (0–2 vs. 3–6) at 90 d	SVM	10-fold cross-validation	35,798	Registry data	f1-score 87.9 ± 0.2% (92.9 ± 0.1%, with follow-up data)	mRS score at 30 d, toilet use degree of dependence	“ “	More severe strokes accounted for most prediction errors
Brugnara et al. (66)	Prediction of mRS score (0–2 vs. 3–6) at 90 d	“Machine-learning models with gradient boosting classifiers”	Not specified	246	Clinical data, radiological data (CT, CTA, CTP, and angiographic images)	AUC 0.856	NIHSS score at 24 h, Premorbid mRS score, Final infarct volume on CT	“ “	Single center, No external validation, Retrospective
Forkert et al. (67)	Prediction of mRS score at 90 d	SVM (specifically the Extended Problem- specific model)	Leave-one-out cross-validation	68	Clinical data, MRI images	•mRS score ± 1: 82.4% accuracy •mRS score 0–2 vs. 3–6: 85.4% accuracy	•L-hemisphere strokes: lesion-based *t*-score sum •Rt-hemisphere strokes: Lesion volume	“ “	No external validation, Retrospective
Monteiro et al. (68)	Prediction of mRS score (0–2 vs. 3–6) at 90 d	RF	10-fold cross-validation	425	Clinical data, CT or MRI images	AUC 0.936 ± 0.34	Baseline NIHSS score, Baseline NIHSS score on subsection 2 (Best gaze, horizontal EOMs)	“ “	Single center, No external validation, Retrospective, Performed worse than non-imaging model
Jang et al. (69)	Prediction of mRS score (>1 vs. >2) at 90 d	XGBoost	3-fold cross-validation and a random search strategy	6,731	Registry data	•mRS >1: AUC 0.84 •mRS >2: AUC 0.87		“ “	Treatment-related factors were not included, No external validation
Hope et al. (70)	Prediction of speech production scores	GPR	Leave-one-out cross-validation	270	Clinical data, Assessments, MRI images	*R*^2^ 0.59	Time post-stroke, Lesion site	Informs patient expectations	Post-stroke imaging obtained over a wide range of times (<1 month to +30 y), No external validation, Retrospective
Lopes et al. (71)	Prediction of cognitive functions at 3 y after minor stroke	Ridge Regression	3-step nested leave-one-out cross-validation, consisting of inner, middle, and outer loops	72	Clinical data, Assessments, functional MRI images	*R*^2^ values for attention, memory, visuospatial functions, and language functions: 0.73, 0.67, 0.55, 0.48	-	“ “	Limited generalizability (mean NIHSS on admission was 1.5 ± 2.2), Retrospective
Sale et al. (72)	Prediction of change in BI score and FIM score during inpatient rehab	SVM	Nested 5-fold cross-validation	55	Clinical biomarker data, Assessments	Discharge cognitive FIM score: MADP 17.55%, RMSE 4.28	Cognitive FIM score upon admission	Informs patient expectations, Facilitates GOC decision making	Small sample size, included hemorrhagic stroke patients
Iwamoto et al. (73)	Prediction of ADL dependence after inpatient rehab	CART method	Not specified	994	Clinical data, Assessments	AUC 0.83	FIM transfer score (≤ 4 or >4)	“ “	Single center, Retrospective
Lin et al. (74)	Prediction of BI score (<60, 60–90, >90) upon discharge from inpatient rehab	LR, RF	5-fold cross-validation	313	Clinical data, Assessments	LR: AUC 0.796, RF: AUC 0.792	BI, IADL, and BBT scores on admission	“ “	Limited generalizability due to aggressive rehab strategy, No external validation
Tozlu et al. (75)	Prediction of post-intervention UE motor impairment in chronic stroke	Elastic net	Nested 10-fold cross-validation with outer and inner loops	102	Clinical data, Assessments	Median *R*^2^ 0.91	Pre-intervention UE-FMA, difference in MT between the affected and unaffected hemispheres	Informs patient expectations, Increases rehabilitation efficiency	Retrospective, No external validation
Stinear et al. (76)	Predicts potential for UE recovery	Cluster analyses	Not applicable	40	Clinical assessments ± neurophysiological assessments and MRI images	Partial η^2^ 0.811	-	“ “	Small sample size, Single center, No external validation

***Section A and B***

*ADL, Activities of daily living; AE, Auto-encoders; AF, Atrial fibrillation; AIS, Acute ischemic stroke; ANN, Artificial neural network; AUC, area under the receiver operating characteristic curve; BBB, blood-brain barrier; BBT, Berg balance test; BI, Barthel Index; CART, Classification and regression tree; CNN, convolutional neural network; CSF, cerebral spinal fluid; CST, Corticospinal tract; CT, computed tomography; CTA, Computerized tomography angiography; CTP, Computerized tomography perfusion; CXR, Chest radiograph; D, days; DCI, delayed cerebral ischemia; DTI, Diffusion Tensor Imaging; DWI, diffusion weighted image; EDH, epidural hematoma; EEG, electroencephalogram; EHR, electronic health record; EOMs, Extra-ocular movements; EVT, endovascular treatment; FBG, Fasting blood glucose; FIM, Functional independence measure; GAM, generalized additive model; GBRT, gradient boosted regression tree; GLM, generalized linear model; GOC, Goals-of-care; GRU, gated recurrent unit; GPR, Gaussian Process model Regression; H, hours; HT, hemorrhagic transformation; IADL, Instrumental activities of daily living scale; ICH, Intracerebral hemorrhage; IVH, intraventricular hemorrhage; KNN, K nearest neighbor; L, Left; LASSO, Least absolute shrinkage and selection operator regression; LR, logistic regression; MADP, Mean absolute percentage deviation; MCA, middle cerebral artery; MCP, matricellular proteins; Min, minutes; MLP, multilayer perceptron; MRC, medical research council; MRI, magnetic resonance imaging; mRS, Modified Rankin Score; MT, motor threshold; NB, naïve bayes; NIHSS, National Institutes of Health Stroke Scale; PHE, perihematomal edema; PPI, Proton pump inhibitor; RF, Random forest; RFNN, Structured Receptive Field Neural Networks; RMSE, Root mean square error; ROI, region of interest; Rt, Right; SAP, Stroke-associated pneumonia; sICH, symptomatic intracranial hemorrhage; SMOTE, synthetic minority oversampling technique; SMR, stepwise multilinear regression; SR-KDA, Kernel Spectral Regression for Discriminant Analysis; SVM, support vector model; SVM-P, support vector machine with polynomial; SVM-R, support vector machine with radial basis function; UE, Upper extremity; UE-FMA, Upper extremity Fugl-Meyer Assessment; XGBoost, Extreme gradient boosting; Yr, year.*

NB: List of ML terms with definitions is provided in **Supplementary Table 1**.

***Section B***

*Many of the listed studies utilize a variety of machine learning (ML)-based approaches. The approach listed on the table is the approach with the optimal result from each individual study.*

* *Phenotype based on Oxfordshire Community Stroke Project (OCSP) (total anterior circulation infarcts, lacunar infarcts, partial anterior circulation infarcts, posterior circulation infarcts)*.

## Methods

We searched PubMed, Google Scholar, Web of Science, and IEEE Xplore^®^ for relevant articles using various combination of the following key words: “machine learning,” “artificial intelligence,” “stroke,” “ischemic stroke,” “hemorrhagic stroke,” “diagnosis,” “prognosis,” “outcome,” “big data,” and “outcome prediction.” Resulting abstracts were screened by all authors and articles were hand-picked for full review based on relevance and scientific integrity. Final article list was reviewed and approved by all authors.

### Machine Learning in Stroke Diagnosis

The time-sensitive nature of stroke care underpins the need for accurate and rapid tools to assist in stroke diagnosis. Over the recent years, the science of brain imaging has vastly advanced with the availability of a myriad of AI based diagnostic imaging algorithms (77). Machine learning is particularly useful in diagnosis of acute stroke with large vessel occlusion (LVO). Various automated methods for detection of stroke core and penumbra size as well as mismatch quantification and detection of vascular thrombi have recently been developed (77). Over the past decade, 13 different companies have developed automated and semi-automated commercially available software for acute stroke diagnostics (Aidoc^®^, Apollo Medical Imaging Technology^®^, Brainomix^®^, inferVISION^®^, RAPID^®^, JLK Inspection^®^, Max-Q AI^®^, Nico.lab^®^, Olea Medical^®^, Qure.ai^®^, Viz.ai^®^, and Zebra Medical Vision^®^) (78). The RapidAI^®^ and Viz.ai^®^ technology have been approved under the medical device category of computer-assisted triage by the United States Food and Drug Administration (FDA). The RAPID MRI^®^ (Rapid processing of Perfusion and Diffusion) software allows for an unsupervised, fully-automated processing of perfusion and diffusion data to identify those who may benefit from thrombectomy based on the mismatch ratio (79). Such commercial platforms available for automatic detection of ischemic stroke and LVO have facilitated rapid treatment decisions. When compared to manual segmentation of lesion volume and mismatch identification from patients enrolled in DEFUSE 2, the RAPID results were found to be well-correlated (*r*^2^ = 0.99 and 0.96 for diffusion and perfusion weighted imaging, respectively) with 100% sensitivity and 91% specificity for mismatch identification (80). Since 2008, the RapidAI^®^ platform has expanded to include other products (Rapid^®^ ICH, ASPECTS, CTA, LVO, CTP, MRI, Angio, and Aneurysm) that assist across the entire spectrum of stroke. Viz LVO^®^ was the first FDA-cleared software to detect and alert clinicians of LVO *via* the “Viz Platform” (81). In a recent single center study with 1,167 CTAs analyzed, Viz LVO^®^ was found to have a sensitivity of 0.81 and a negative predictive value of 0.99 with an accuracy of 0.94 (82).

Other areas of stroke diagnostics that have seen an increase in attention over the past decade are the identification of intracerebral hemorrhage (ICH) and patients at risk for delayed cerebral ischemia in the setting of aneurysmal subarachnoid hemorrhage (aSAH). While most studies tend to have good accuracy in detecting an ICH there is more variability in subclassification and measurements of hematoma volume. A summary of recent publications on ML in stroke diagnosis is presented in Table 1 (Section A).

### Machine Learning in Stroke Outcome Prediction

Despite recent advances in stroke care, it remains the second leading cause of death and disability world-wide (4, 83). Although acute stroke diagnosis and determination of the time of stroke onset are the initial steps of comprehensive stroke management, clinicians are also often charged with the task of determining stroke outcomes. These outcomes range from discrete radiological outcomes (e.g., final infarct volume, the likelihood of hemorrhagic transformation, etc.), the likelihood of morbidity (e.g., stroke-associated pneumonia) and mortality, and various measures of functional independence (e.g., mRS score, Barthel Index score, cognitive, and language function, etc.).

Prognostication after an acute brain injury is notoriously challenging, particularly within the first 24–48 h (84). However, a clinician may be called upon to provide estimates of a patient's short-term and long-term mortality and degree of functional dependence to assist with decision-making regarding the intensity of care (e.g., use of thrombolytics or endovascular treatment, intubation, code status, etc.) (60, 64, 66, 67, 69, 70, 72–76). Like all medical emergencies, it is incumbent upon the stroke clinician to ensure that all care provided is concordant with an individual patient's goals (85). For example, a surrogate decision-maker may decline to reverse a patient's longstanding “do not intubate” order to facilitate mechanical thrombectomy if the clinician predicts the patient has a high likelihood of functional dependence or short-term mortality. Hence, accuracy in outcome prediction is critical in guiding management of our patients.

Determining a patient's likelihood of developing symptomatic intracranial hemorrhage (sICH) is of obvious, immediate value in acute stroke management in determining candidacy for thrombolytic therapy or endovascular treatment. Historically, clinician-based prognostication tools to predict the risk of symptomatic intracranial hemorrhage after IV thrombolysis, such as the SEDAN (Sugar, Early Infarct signs, Dense cerebral artery sign, Age, and NIHSS) and HAT (Hemorrhage After Thrombolysis) scores have been used to predict the risk of symptomatic intracranial hemorrhage after IV thrombolysis (23). Advances in ML and DL have allowed for the development of more accurate models which outperform the traditional SEDAN and HAT scores (23, 54, 55). Similarly, the ability to predict final infarct volume and the likelihood of the development of malignant cerebral edema have important treatment implications and remain a significant focus of ML in stroke (26, 51–53).

In patients with intracerebral hemorrhage (ICH), the ICH-score is one of the most widely used clinical prediction scores (85–88). Although ML technology for outcome prediction has rapidly advanced for ischemic stroke, recent ML studies predicting functional outcomes after ICH have also demonstrated high-discriminating power (63, 89). A recent study by Sennfält et al. tracked long-term functional dependence and mortality after an acute ischemic stroke of more than 20,000 Swedish patients (90). The 30-day mortality rate was 11.1%. At 5 years, 70.6% of ischemic stroke patients were functionally dependent (defined as mRS score of ≥3) or had died (5-year mortality rate of 50.6%). These sobering outcomes partially account for the development of many stroke prognostic models over the years, which frequently serve as benchmarks in stroke research. Recently, Matsumoto et al. compared the performance of six existing stroke prognostic models for predicting poor functional outcomes and in-hospital mortality with linear regression or decision tree ensemble models (59). The novel prediction models performed slightly better than the conventional models in predicting poor functional outcomes (AUC 0.88–0.94 vs. AUC 0.70–0.92) but were equivalent or marginally worse in predicting in-hospital death (AUC 0.84–0.88 vs. AUC 0.87–0.88). Many such stroke prediction models have emerged over the recent years. An overview of ML based automated algorithms for stroke outcome prediction is provided in Table 1 (Section B).

## Discussion

In recent years, some DL algorithms have approached human levels of performance in object recognition (91). One of the greatest strengths of ML is its ability to endlessly process data and tirelessly perform an iterative task. Further, creation of a ML model can be performed much faster (i.e., in a matter of 5–6 days compared with 5–6 months or even years) than traditional computer-aided detection and diagnosis (CAD) (92). which makes ML an attractive field for computer experts and scientists. Several ML tools are currently in use including the FDA-approved ML algorithms previously discussed for rapid stroke diagnosis which have significantly enhanced the workflow of acute ischemic stroke patients.

Despite the prolific advent of new and improved ML algorithms with increasing clinical applications, it is important to recognize that computer-based algorithms are only as good as the data used to train the models. For a reliable algorithm, it is important to develop well-defined training, validation, and testing sets. Testing should be done on a diverse set of data points reflective of a real-world scenario. Overfitting can be an issue in ML algorithms when the model is trained on a group of highly-selected, specific features, which when tested on a larger dataset with varied features, fails to perform adequately. Similarly, underfitting can occur when a model is oversimplified with generalized feature selection in the training set which then becomes unable to capture the relevant features within a complex pattern of a larger or more diverse testing set. The aphorism “garbage in, garbage out” remains true as the use of inadequate or unvalidated data points (e.g., unverified clinical reports from electronic health record) in the training set can lead to poor performance of the ML algorithm in the testing set. Hence, it is important to note that the algorithmic decision-making tools do not guarantee accurate and unbiased interpretation compared to established logistic regression models (56, 59, 93). Comparisons to well-established models should be standard when developing new ML algorithms given the high cost associated with ML (e.g., the time required to collect data, train the model, perform internal and external validations, cost of reliable and secure data storage, etc.) (94). Specifically, as it relates to diagnostics there are a myriad of considerations that must be taken into account. Not only should the algorithm provide accurate information quickly, but it should have the ability to integrate into the electornic health record (EHR) to improve end user experience and efficiency in workflow. Programs such as RAPID^®^, Viz.ai^®^, and Brainomix^®^ have started to successfully integrate into the EHR, which has helped expedite acute stroke diagnosis and triage process. One of the major technical challenges of ML include the ability to develop an algorithm with a “reasonable” detection rate of pathology without an excessive rate of false-positives. For example, there are notable discrepancies among various ML studies for ICH diagnosis, with varying accuracy depending on the type of ICH (e.g., spontaneous ICH, SDH, aSAH, or IVH). Overfitting and underfitting of the model could lead to poor applicability and therefore, image preprocessing with meticulous feature selection is necessary. Furthermore, the “black-box” nature of ML precludes the clinicians from identifying and addressing biases within the algorithms (95, 96). Hence, proper external validation is necessary to ensure generalizability of the algorithm in diverse clinical scenarios.

For stroke prediction, most existing ML algorithms utilize dichotomized outcomes. Functional outcome is frequently defined as “good” when mRS score is 0–2 and “poor” when mRS score is 3–6 by convention and IS studies often measure mRS score at 90 days after stroke (64–69, 97). However, the medical community is increasingly embracing patient-centered outcomes. People are starting to recognize the need for longitudinal patient follow-up given potential for functional improvement beyond conventional norms of 90 days (98). Once patient-centered outcomes are clinically validated (e.g., MRS cutoff of 0–2 vs. 3–6, 0–3 vs. 4–6, or 0–4 vs. 5–6), new ML algorithms incorporating such outcomes would be increasingly helpful to the clinicians. The use of high-yield, ML programs using patient-centered outcomes could ease the commonplace but challenging discussions of the anticipated quality of life and the risk of long-term dependency or death before deciding on a patient's goals-of-care. It is however important to apply caution while using ML algorithms for outcome prediction as patient demographics and clinical practice continue to evolve and updates to the ML algorithms would be necessary to remain applicable to evolving patient populations and clinical standards. Additionally, developers often retrieve data from existing datasets (e.g., clinical trial data) with its inherent biases including selection bias, observer bias and other confounders (e.g., withdrawal of life supporting therapy may be more common in older patients with large hemispheric stroke compared to younger patients, which could confound outcome prediction in older patients compared to younger ones).

Overall, compared to other diseases such as Alzheimer's disease, there is a relative paucity of large, high-quality datasets within stroke. Some limitations that have stymied the development of large, open-access stroke registries include the need for data-sharing agreements, patient privacy concerns, high costs of data storage and security, arbitration of quality control of the input data, etc. (95). Cohesive and collaborative efforts across hospital systems, regions, and nations with data acquisition and harmonization is needed to improve future ML-based programs in stroke. With adoption of EHR systems, healthcare data is rapidly accumulating with an estimated over 35 zettabytes of existing healthcare data! (99). Adoption of AI and ML algorithms allow us to efficiently process the plethora of information that surround us every day. Nonetheless, as we continue to adapt to this evolving landscape of medical practice surrounding big data, clinicians need to remain aware of the limitations of this modern day “black box” magic.

## Conclusion

The emerging ML technology has rapidly integrated into multiple fields of medicine including stroke. Deep learning has significantly enhanced practical applications of ML and some newer algorithms are known to have comparable accuracy to humans. However, the diagnosis and prognosis of a disease, including stroke, is highly intricate and depends on various clinical and personal factors. The development of optimal ML programs requires comprehensive data collection and assimilation to improve diagnostic and prognostic accuracy. Given the “black box” or cryptic nature of these algorithms, it is extremely important for the end-user (i.e., clinicians) to understand the intended use and limitations of any ML algorithm to avoid inaccurate data interpretation. Although ML algorithms have improved stroke systems of care, blind dependence on such computerized technology may lead to misdiagnosis or inaccurate prediction of prognostic trajectories. At the current state, ML tools are best used as “aids” for clinical decision making while still requiring oversight to address relevant clinical aspects that are overlooked by the algorithm.

## Author Contributions

SM: substantial contributions including conception and design of the work, literature review, interpretation and summarization of data, drafting the complete manuscript, revising it critically for important intellectual content, and final approval of the manuscript to be published. MD and KS: contribution including conception and design of the work, literature review, interpretation and summarization of the data, drafting of critical portion of the manuscript, critical revision for important intellectual content, and final approval of the manuscript. All authors contributed to the article and approved the submitted version.

## Funding

This article was supported by the Virginia Commonwealth University, Department of Neurology.

## Conflict of Interest

The authors declare that the research was conducted in the absence of any commercial or financial relationships that could be construed as a potential conflict of interest.

## Publisher's Note

All claims expressed in this article are solely those of the authors and do not necessarily represent those of their affiliated organizations, or those of the publisher, the editors and the reviewers. Any product that may be evaluated in this article, or claim that may be made by its manufacturer, is not guaranteed or endorsed by the publisher.
