# Supplementary material for: Machine Learning in Action: Stroke Diagnosis and Outcome Prediction
Source: Front Neurol. 2021 Dec 6;12:734345. doi: 10.3389/fneur.2021.734345 (PMC8685212; doi:10.3389/fneur.2021.734345)
Supplement: Supplementary file 1 [file Table_1.DOC]

Supplementary Table 1. Glossary of select machine learning terminology

| **Term** | **Definition** |
| --- | --- |
| Adaptive Boosting, (Adaboost) | An algorithm used in combination with others to convert a set of weak classifiers into a strong one |
| Adaptive network based fuzzy inference system (ANFIS) | A fuzzy Sugeno model put in the framework of adaptive systems to facilitate learning and adaptation. |
| Artificial Neural Network (ANN) | A computational model based on a collection of connected units or nodes called artificial neurons, which loosely model the neurons in a biological brain |
| Auto-encoders | An unsupervised learning technique which use ANNs to learn efficient codings (i.e., a representation) for a set of unlabeled data |
| Bayesian Network (BN) | A type of probabilistic graphical model that uses Bayesian inference for probability computations |
| Bootstrapping | Resampling multiple new datasets with replacement from the original data set |
| Calibration | Adjusting the predicted probability from the model to more closely match the observed probability in the test set |
| Classification and  regression tree (CART) | A predictive model which uses a binary tree design to explain how an outcome variable's values can be predicted based on other values |
| Convolutional Neural  network (CNN) | A class of feedforward neural networks which use at least one convolutional layer to filter inputs for useful information |
| Cross-validation (CV) | A model validation technique for assessing how the results of a statistical analysis (model) will generalize to an independent data set |
| Decision Tree | A tree with a set of hierarchical decisions which eventually gives a final decision |
| Discrimination | The ability of a model to separate individual observations in multiple classes |
| Feature | Predictor or independent variable in a ML model |
| Feature selection | Variable selection or attribute selection |
| Gaussian Process  model regression | A probabilistic supervised ML framework used for regression and classification tasks that make predictions incorporating prior knowledge and provide uncertainty measures rather than predictions |
| Generalization ability | The ability of a model to generalize the learned pattern to new data |
| Generalized additive  model | An additive modeling technique where the impact of the predictive variables is captured through smooth functions which may be linear or nonlinear |
| Generalized Linear  model | A generalization of least squares regression models, which are based on Gaussian noise, to other types of models based on other types of noise |
| Gradient-boosted regression  tree | A ML technique that uses an ensemble of weak prediction models to perform regression and classification tasks |
| Hyperparameters | Configurations of a model which are often selected and set before training the model |
| Imputation | The process of replacing missing data with substituted values |
| k-nearest neighbors (kNN) | A type of instance-based learning, where the prediction is only approximated locally with the k nearest neighbors |
| Kernel Spectral  Regression for  Discriminant Analysis  (SR-KDA) | A means of saving computational cost by casting discriminant analysis into a regression framework |
| Least Absolute Shrinkage and Selection Operator (LASSO) | A regression technique that performs both variable selection and regularization |
| Leave-one-center-out CV | A performance measurement approach that uses observations from one center as the validation set and the remaining observations as the training set |
| Leave-one-out CV | A performance measurement approach that uses one observation as the validation set and the remaining observations as the training set |
| Logistic regression | A regression model that uses a sigmoid function to convert a linear model's raw prediction into a value between 0 and 1 |
| Mean absolute percentage  deviation | The mean of the absolute percentage errors of forecasts, which is a measure of the prediction accuracy of a forecasting model |
| Missing data mechanism | Three missing-data mechanisms: missing completely at random (MCAR), missing at random (MAR), and missing not at random (MNAR) |
| Naïve Bayes (NB) | A family of simple "probabilistic classifiers" based on applying Bayes' theorem with strong (naïve) independence assumptions between the features |
| Over-fitting | A model corresponds too closely or exactly to a particular set of data, and may fail to fit new data |
| Parameters | Coefficients of a model that need to be learned from the data |
| Random Forest (RF) | An ensemble learning method that uses a multitude of decision trees |
| Region of interest  (ROI) | A proposed region from an original image which is widely used in object detection tasks using CNNs |
| Root mean square  error | The square root of the mean squared error, which is the average squared loss per example |
| Stepwise multilinear  regression | A method of regressing multiple variables repeatedly which allows for the identification of the weakest correlated variable thereby allowing improvement of the model by only retaining the variables which describe the distribution best |
| Structured Receptive Field Neural Networks | A CNN that treats images as functions in Scale-space, where the learned convolution kernels become functions as well |
| Super learner | A stacking algorithm using cross-validated predictions of other models and assigning weights to these predictions to optimize the final prediction |
| Supervised learning | A subgroup of ML models that requires both predictors and outcomes (labels) |
| Support Vector Machine (SVM) | A supervised classifier that seeks to find the best hyperplane to separate the data |
| Support vector model  with polynomial | The addition of a polynomial kernel to an SVM allows for development of a hyperplane in a very high dimensional feature space |
| Support vector model  with radial basis  function (RBF) | RBF kernel is a function whose value depends on the distance from the origin or from some point that is used to calculate the similarity or angular distance between two points |
| Synthetic minority  oversampling  technique (SMOTE) | A means of creating synthetic examples for the minority class to create a more balanced data set which will more effectively allow a model to learn the decision boundary |
| Testing | A validation set used for testing the model |
| Training | The learning process of the data pattern by a model |
| Unsupervised learning | A subgroup of ML models meant to find previously unknown patterns in data without pre-existing labels |
| Validation | A process used, as part of training, to evaluate the quality of a machine learning model using the validation set. |
| Xgboost | A decision-tree-based ensemble ML algorithm that uses a gradient boosting framework |

Adapted from “[Table 1. Notations of special machine learning terms](https://journals.plos.org/plosone/article?id=10.1371/journal.pone.0234722)” by [Wang W, et al^100.^](http://orcid.org/0000-0002-1879-7332) and [Machine Learning Glossary](https://developers.google.com/machine-learning/glossary#w)^101^ by [Google Developers](https://developers.google.com/machine-learning/crash-course) which are both licensed under the [Creative Commons Attribution 4.0 License](https://creativecommons.org/licenses/by/4.0/).
